# Adaptive Control of a Vibratory Angle Measuring Gyroscope

**DOI:** 10.3390/s100908478

**Published:** 2010-09-09

**Authors:** Sungsu Park

**Affiliations:** Department of Aerospace Engineering, Sejong University, 98, Gunja-dong, Kwangjin-gu, Seoul 143-747, Korea; E-Mail: sungsu@sejong.ac.kr; Tel.: +82-2-3408-3769; Fax: +82-2-3408-4333.

**Keywords:** angle measurement, vibratory gyroscope, trajectory following, adaptive control

## Abstract

This paper presents an adaptive control algorithm for realizing a vibratory angle measuring gyroscope so that rotation angle can be directly measured without integration of angular rate, thus eliminating the accumulation of numerical integration errors. The proposed control algorithm uses a trajectory following approach and the reference trajectory is generated by an ideal angle measuring gyroscope driven by the estimate of angular rate and the auxiliary sinusoidal input so that the persistent excitation condition is satisfied. The developed control algorithm can compensate for all types of fabrication imperfections such as coupled damping and stiffness, and mismatched stiffness and un-equal damping term in an on-line fashion. The simulation results show the feasibility and effectiveness of the developed control algorithm that is capable of directly measuring rotation angle without the integration of angular rate.

## 1. Introduction

MEMS vibratory gyroscopes are typically designed to measure the angular rate [1]. In order to obtain the rotation angle, the measured angular rate with respect to time must be integrated. The integration process, however, causes the rotation angle to drift over time and therefore the angle error to diverge quickly due to the presence of bias and noise in the angular rate signal. This error accumulation problem is more severe for low cost MEMS rate gyroscopes. Thus, for eliminating the accumulation of numerical integration errors, it is required a novel angle measuring gyroscope capable of direct measurement of rotation angle without integration of the angular rate.

MEMS vibratory gyroscopes can conceptually operate in the rotation angle measurement mode, where integration is done mechanically rather than electronically [2]. This angle measurement mode works on the same principles as a Foucault Pendulum. When an isotropic oscillator of the gyroscope is allowed to freely oscillate, the precession of the straight line of oscillation provides a measure of the rotation angle [3]. For freely oscillating, the natural frequencies of oscillation of the two vibrating modes must be the same and the modes are un-damped. Ideally, the stiffness of suspension and the damping of the gyroscope should be perfect isotropic, the vibrating modes of a MEMS gyroscope are supposed to remain mechanically decoupled, and the output of the gyroscope should be sensitive to only rotation. In practice, however, the tolerance of manufacturing precision does not allow it, and fabrication defects and environment variations are always present, resulting in a mismatch of the frequencies of oscillation for the two vibrating modes and the presence of linear dissipative forces with damping coefficients [4]. These fabrication imperfections are major factors that limit realization of an angle measuring gyroscope.

If a MEMS angle measuring gyroscope can be developed, it will open up new market opportunities and applications in the area of small, low-cost and medium-performance inertial devices. For example, the angle measuring gyroscope can directly measure the yaw angle which is not affected by external interference such as magnetic disturbances. The angle measuring gyroscope can also be combined with regular rate gyroscopes, accelerometers and/or magnetometers to improve accuracy and robustness of attitude measurements. Moreover, three-axis angle measuring gyroscope constitutes an attitude reference system (ARS) which can greatly reduce the size and cost of current ARS.

Although a MEMS angle measuring gyroscope is a promising sensor, it is not developed yet, and even in the literature, very few control algorithms have been reported for realizing angle measuring gyroscopes [2,3,5–8], while most published control algorithms deal with rate gyroscopes [9–12]. This is because angle measurement is much more challenging task than the angular rate measurement. For angle measurement, the oscillator of the vibratory gyroscope should be initiated and maintained as the straight line in “inertial frame”, rather than in gyro frame as in the rate gyroscope.

Along with the efforts of robust structure design to the fabrication imperfections, active compensations are required to null out the remaining imperfections and environment variations during the operation. Friedland and Hutton [5] suggested the use of a vibratory gyroscope for measuring rotation angle. A composite nonlinear feedback control is reported in [2,6,7], where the energy control and angular momentum control are developed based on the analytic results of [5]. However, their energy control relies on the equal damping assumption, and the angular momentum control is vulnerable to interference with the Coriolis acceleration. Another composite nonlinear feedback control is proposed in [8], where the stability of the controlled system is not proven. Park *et al*. [3] also present a control algorithm that consists of energy controls and mode tuning controls to compensate for mismatched stiffness and damping. The stability is theoretically proven. But they assume zero coupled damping and their approach requires a calibration for damping ratio of two axes prior to the normal operation.

In this paper, we present a new adaptive control algorithm for realizing angle measuring gyroscopes. Compared to the previous works [2–3,5–8], the proposed algorithm does not need a calibration session, but it can compensate for all types of fabrication imperfections in an on-line fashion such as coupled damping and stiffness, which normally cause quadrature errors, and mismatched stiffness and un-equal damping term, and make a non-ideal gyroscope behave like an ideal angle measuring gyroscope.

## 2. Dynamics of a Vibratory Gyroscope

**Figure 1.**
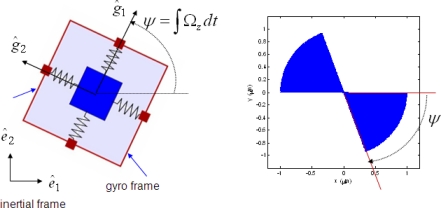
**(a)** Model of ideal gyroscope. **(b)** Precession of the proof mass in gyro frame.

The dynamics of an ideal vibratory angle measuring gyroscope is defined as follows:

(1) $$\begin{array}{l} \ddot{x}+\omega_{0}^{2}x=2\Omega_{z}\dot{y}\\ \ddot{y}+\omega_{0}^{2}y=-2\Omega_{z}\dot{x} \end{array}$$

where *x* are *y* the coordinates of the proof mass relative to the gyro frame. Equation (1) presents a two degree-of-freedom (2-DOF) pure spring-mass system with the same natural frequency *ω*_0_ in both axes, which is oscillating on a rotating gyro frame with a constant angular rate Ω*_z_* as shown in Figure 1. If the line of oscillation of the mass with amplitude *M* is initially aligned with the *ê*_1_ axis of the inertial frame, then the solution of Equation (1) is given by:

(2) $$\begin{array}{l} x(t)=M\text{cos}(\Omega_{z}t)\text{sin}(\omega_{0}t)\\ y(t)=-M\text{sin}(\Omega_{z}t)\text{sin}(\omega_{0}t) \end{array}$$

The rotation angle (*ψ* = Ω*_z_t*) can be calculated with Equation (2) by measuring position of the proof mass, *x* and *y*, in the gyro frame. The behavior of ideal gyroscope is plotted in Figure 1(b) and shows that the precession of the line of oscillation of the mass can provide a measure of the rotation angle.

A physical angle measuring gyroscope can be implemented by the 2-DOF mass-spring-damper system whose proof mass is suspended by spring flexure anchored at the gyro frame. Considering fabrication imperfections and damping, a realistic model of a z-axis gyroscope is described as follows:

(3) $$\begin{array}{l} \ddot{x}+d_{xx}\dot{x}+d_{xy}\dot{y}+\omega_{x}^{2}x+\omega_{xy}y=f_{x}+2\Omega_{z}\dot{y}\\ \ddot{y}+d_{xy}\dot{x}+d_{yy}\dot{y}+\omega_{xy}x+\omega_{y}^{2}y=f_{y}-2\Omega_{z}\dot{x} \end{array}$$

where *d_xx_* and *d_yy_* are damping, *ω_x_* and *ω_y_* are natural frequencies of the x- and y-axis, *d_xy_* and *ω_xy_* are coupled damping and frequency terms, and *f_x_* and *f_y_* are the specific control forces applied to the proof mass in *ĝ*_1_ and *ĝ*_2_ axis of the gyro frame, respectively. The coupled damping and frequency terms, called quadrature errors, comes mainly from asymmetries in suspension structure and misalignment of sensors and actuators. Therefore, the control problem of angle measuring gyroscope is to determine control laws for *f_x_* and *f_y_* which make a non-ideal gyroscope (3) behaves like the ideal gyroscope (1).

## 3. Adaptive Control Algorithm

In this section, we present an adaptive controller to realize an angle measuring gyroscope. The basic idea of the adaptive control approach is to treat both the angular rate and the fabrication imperfections as unknown gyroscope parameters, and these are estimated using a parameter adaptation algorithm (PAA). In adaptive control problems, the persistent excitation condition is an important factor to estimate the unknown parameters correctly. To solve this problem, a trajectory following approach is used. The reference trajectory that the gyroscope must follow should be generated such that the persistent excitation condition is met.

A reference trajectory may be generated by an ideal angle measuring gyroscope (1). However, the dynamics of an ideal gyroscope is not sufficiently exciting for parameter identification and moreover the angular rate Ω*_z_* is not known. Instead, we propose that a reference trajectory is generated by an ideal angle measuring gyroscope driven by estimate of angular rate Ω̂*_z_* and the auxiliary sinusoidal input *f_xm_*, *f_ym_* as follows:

(4) $$\begin{array}{l} {\ddot{x}}_{m}+\omega_{0}^{2}x_{m}=2{\hat{\Omega }}_{z}{\dot{y}}_{m}+f_{xm}\\ {\ddot{y}}_{m}+\omega_{0}^{2}y_{m}=-2{\hat{\Omega }}_{z}{\dot{x}}_{m}+f_{ym} \end{array}$$

where *f_xm_* = *f_ym_* = *F*_0_ sin(*ω_f_t*) and *ω*_0_ ≠ *ω_f_*. The role of the auxiliary sinusoidal input is to increase the complexity of the internal dynamics of the gyroscope so that its response is persistently exciting and all fabrication imperfections and environmental variations can be identified and compensated for in an on-line fashion. Note that compared to the dynamics of an ideal angle measuring gyroscope, the dynamics of reference Gyroscope Model (4) is driven by two sources, the estimate of angular rate and the auxiliary sinusoidal input. Thus for rotation angle calculation, the effect of auxiliary sinusoidal input should be removed. The detailed angle calculation method will be discussed in Section 4.

Now, let us rewrite the dynamics of non-ideal Gyroscope (3) and the reference Gyroscope Model (4) as follows:

(5) $$\;\ddot{q}+\omega_{0}^{2}q=f-D\dot{q}-Rq-2\Omega \;\dot{q}$$

where:

$$\begin{array}{l} q={\left[x\;y\right]}^{T},\;f={\left[f_{x}\;f_{y}\right]}^{T},\;D=\left[\begin{array}{cc} d_{xx} & d_{xy}\\ d_{xy} & d_{yy} \end{array}\right],\;\Omega =\left[\begin{array}{cc} 0 & -\Omega_{z}\\ \Omega_{z} & 0 \end{array}\right],\;R=\left[\begin{array}{cc} \Delta \omega_{x} & \omega_{xy}\\ \omega_{xy} & \Delta \omega_{y} \end{array}\right],\\ \;\;\;\;\;\;\;\;\;\;\;\;\;\;\;\;\;\;\;\;\;\;\;\;\Delta \omega_{x}=\omega_{x}^{2}-\omega_{0}^{2},\;\Delta \omega_{y}=\omega_{y}^{2}-\omega_{0}^{2} \end{array}$$

and:

(6) $${\ddot{q}}_{m}+\omega_{0}^{2}q_{m}=-2\hat{\Omega }{\dot{q}}_{m}+f_{m}$$

where:

$$q_{m}={\left[x_{m}\;y_{m}\right]}^{T},\;f_{m}={[f_{mx}\;f_{my}]}^{T},\;\hat{\Omega }=\left[\begin{array}{cc} 0 & -{\hat{\Omega }}_{z}\\ {\hat{\Omega }}_{z} & 0 \end{array}\right].$$

Then the control problem is formalized as follows: given the Equation (5) with unknown constant parameters *D*, *R* and Ω, determine the control law *f* such that the dynamics of Equation (5) follows that of Equation (6), and *D*, *R* and Ω are estimated correctly.

Defining the trajectory error as *e_p_* = *q* – *q_m_*, if the control law *f* is chosen to be:

(7) $$f=f_{0}+f_{m}+\hat{D}{\dot{q}}_{m}+\hat{R}q_{m}$$

where *f*_0_ will be subsequently defined, and *D̂* and *R̂* are estimates of *D* and *R* respectively, then the trajectory error dynamics becomes:

(8) $${\ddot{e}}_{p}+(D+2\Omega ){\dot{e}}_{p}+{Ke}_{p}=f_{0}+\tilde{D}{\dot{q}}_{m}+\tilde{R}q_{m}+2\tilde{\Omega }\;{\dot{q}}_{m}$$

where:

$$\tilde{D}=\hat{D}-D,\;\tilde{R}=\hat{R}-R,\;\tilde{\Omega }=\hat{\Omega }-\Omega ,\;K=\left[\begin{array}{cc} \omega_{x}^{2} & \omega_{xy}\\ \omega_{xy} & \omega_{y}^{2} \end{array}\right]$$

Considering the following Lyapunov function candidate:

(9) $$\;V=\frac{1}{2}\left(\gamma_{1}{\dot{e}}_{p}^{T}{\dot{e}}_{p}\;+\;\gamma_{1}e_{p}^{T}{Ke}_{p}\;+\;tr\left\{\gamma_{R}^{-1}\tilde{R}{\tilde{R}}^{T}+\gamma_{D}^{-1}\tilde{D}{\tilde{D}}^{T}+\gamma_{\Omega }^{-1}\tilde{\Omega }{\tilde{\Omega }}^{T}\right\}\right)$$

where *γ*_(·)_ are positive constants and *tr*{*A*} defines the trace of the matrix *A*. The time derivative of Lyapunov function along the trajectory of the Equation (8) is:

(10) $$\begin{array}{c} \dot{V}=-\gamma_{1}{\dot{e}}_{p}^{T}\left(D+2\Omega \right){\dot{e}}_{p}+\gamma_{1}{\dot{e}}_{p}^{T}\left(f_{0}+\tilde{D}{\dot{q}}_{m}+\tilde{R}q_{m}+2\tilde{\Omega }{\dot{q}}_{m}\right)\\ +\;tr\left\{\gamma_{R}^{-1}\tilde{R}{\dot{\tilde{R}}}^{T}+\gamma_{D}^{-1}\tilde{D}{\dot{\tilde{D}}}^{T}+\gamma_{\Omega }^{-1}\tilde{\Omega }{\dot{\tilde{\Omega }}}^{T}\right\}\; \end{array}$$

If *f*_0_ is chosen to be:

(11) $$f_{0}=-\gamma_{1}(\dot{q}-{\dot{q}}_{m})=-\gamma_{1}{\dot{e}}_{p}$$

then the Equation (10) becomes:

(12) $$\begin{array}{c} \dot{V}=-\gamma_{1}{\dot{e}}_{p}^{T}\left(\gamma_{1}I+D+2\Omega \right){\dot{e}}_{p}\\ +\;\mathrm{tr}\left\{\tilde{D}\left(\gamma_{D}^{-1}{\dot{\hat{D}}}^{T}-\;\frac{1}{2}{\dot{q}}_{m}f_{0}^{T}\;-\;\frac{1}{2}f_{0}{\dot{q}}_{m}^{T}\right)\;+\;\tilde{R}\left(\gamma_{R}^{-1}{\dot{\hat{R}}}^{T}-\;\frac{1}{2}q_{m}f_{0}^{T}\;-\;\frac{1}{2}f_{0}q_{m}^{T}\right)\;+\;\tilde{\Omega }\left(\gamma_{\Omega }^{-1}{\dot{\hat{\Omega }}}^{T}-\;{\dot{q}}_{m}f_{0}^{T}\;+\;f_{0}{\dot{q}}_{m}^{T}\right)\right\}\; \end{array}$$

where *I* is an identity matrix. Therefore, the parameter adaptation laws:

(13) $$\begin{array}{l} \dot{\hat{R}}=\frac{1}{2}\gamma_{R}\left(f_{0}q_{m}^{T}+q_{m}f_{0}^{T}\right)\\ \dot{\hat{D}}=\frac{1}{2}\gamma_{D}\left(f_{0}{\dot{q}}_{m}^{T}+{\dot{q}}_{m}f_{0}^{T}\right)\\ \dot{\hat{\Omega }}=\gamma_{\Omega }\left(f_{0}{\dot{q}}_{m}^{T}-{\dot{q}}_{m}f_{0}^{T}\right) \end{array}$$

lead to $\dot{V}\leq -\gamma_{1}^{2}{\dot{e}}_{p}^{T}{\dot{e}}_{p}\leq 0$.

**Theorem 1** (Stability)

With control laws (7), (11) and PAA (13), the trajectory error *e_p_* and its time derivatives *ė_p_*, *ë_p_* converge to zero.

*Proof*:

For convenience, the regressor *W*(*q_m_*, *q̇_m_*) is introduced as follows:

$$W^{T}(q_{m},{\dot{q}}_{m})\tilde{\theta }=\tilde{D}{\dot{q}}_{m}+\tilde{R}q_{m}+2\tilde{\Omega }{\dot{q}}_{m}$$

where *θ̃* = *θ̂* – *θ* is the parameter estimation error and:

$$\begin{array}{l} W^{T}(q_{m},{\dot{q}}_{m})=\left[\begin{array}{ccccccc} x_{m} & y_{m} & 0 & {\dot{x}}_{m} & {\dot{y}}_{m} & 0 & -2{\dot{y}}_{m}\\ 0 & x_{m} & y_{m} & 0 & {\dot{x}}_{m} & {\dot{y}}_{m} & 2{\dot{x}}_{m} \end{array}\right]\\ {\tilde{\theta }}^{T}=[\Delta {\tilde{\omega }}_{x}\;{\tilde{\omega }}_{xy}\;\Delta {\tilde{\omega }}_{y}\;{\tilde{d}}_{xx}\;{\tilde{d}}_{xy}\;{\tilde{d}}_{yy}\;{\tilde{\Omega }}_{z}] \end{array}$$

The error equations are:

(14) $$\begin{array}{l} {\ddot{e}}_{p}+(\gamma_{1}I+D+2\Omega ){\dot{e}}_{p}+{Ke}_{p}=W^{T}(q_{m},{\dot{q}}_{m})\tilde{\theta }\\ \dot{\tilde{\theta }}=-\Gamma W(q_{m},{\dot{q}}_{m})\gamma_{1}{\dot{e}}_{p} \end{array}$$

where:

$$\Gamma =\mathrm{diag}\left\{\gamma_{R},\frac{1}{2}\gamma_{R},\;\gamma_{R},\;\gamma_{D},\;\frac{1}{2}\gamma_{D},\;\gamma_{D},\;\frac{1}{2}\gamma_{\Omega }\right\}$$

Since the time derivative of the Lyapunov function is negative semi-definite, all elements of the Lyapunov function are bounded. Taking one more derivative of Equation (12) gives:

$$\ddot{V}=-2\gamma_{1}{\dot{e}}_{p}^{T}(\gamma_{1}I+D+2\Omega )\left(-(\gamma_{1}I+D+2\Omega ){\dot{e}}_{p}-{Ke}_{p}+W^{T}(q_{m},{\dot{q}}_{m})\tilde{\theta }\right)$$

Because *e_p_*, *ė_p_* and *θ̃* are bounded, *V̈* is also bounded. Therefore, by Barbalat’s lemma [13], *V̇* → 0, and this gives *ė_p_* → 0. To prove *ë_p_* → 0, from the error Equation (14), we obtain:

$${\dddot{e}}_{p}=-(\gamma_{1}I+D+2\Omega ){\ddot{e}}_{p}-{K\dot{e}}_{p}+W^{T}({\dot{q}}_{m},{\ddot{q}}_{m})\tilde{\theta }+W^{T}(q_{m},{\dot{q}}_{m})\dot{\tilde{\theta }}$$

Since *e⃛_p_* is bounded, *ë_p_* → 0. Now, to analyze the behavior of *e_p_*, we integrate the tracking error:

$$\begin{array}{l} \int_{0}^{\infty }{Ke}_{p}dt=-\int_{0}^{\infty }{\ddot{e}}_{p}dt-\int_{0}^{\infty }(\gamma_{1}I+D+2\Omega ){\dot{e}}_{p}dt+\int_{0}^{\infty }W^{T}(q_{m},{\dot{q}}_{m})\tilde{\theta }dt\\ \;\;\;\;\;\;\;\;\;\;\;\;\;\;\;\;\;\;\;\;=-{{\dot{e}}_{p}|}_{0}^{\infty }-(\gamma_{1}I+D+2\Omega ){e_{p}|}_{0}^{\infty }+{{\bar{W}}^{T}(q_{m},{\dot{q}}_{m})\tilde{\theta }|}_{0}^{\infty }-\int_{0}^{\infty }{\bar{W}}^{T}(q_{m},{\dot{q}}_{m})\dot{\tilde{\theta }}dt \end{array}$$

where *W̄^T^* (*q_m_*, *q̇_m_*) = ∫ *W^T^* (*q_m_*, *q̇_m_*)*dt*. Thus,

$$\left\|\int_{0}^{\infty }{Ke}_{p}dt\right\|\leq \left\|{{\dot{e}}_{p}|}_{0}^{\infty }+(\gamma_{1}I+D+2\Omega ){e_{p}|}_{0}^{\infty }+{{\bar{W}}^{T}(q_{m},{\dot{q}}_{m})\tilde{\theta }|}_{0}^{\infty }\right\|+\left\|{\bar{W}}^{T}(q_{m},{\dot{q}}_{m})\right\|\cdot \left\|{\tilde{\theta }|}_{0}^{\infty }\right\|$$

Again, the integral of *e_p_* is bounded, *e_p_* → 0. Since *e_p_* → 0, *ė_p_* → 0 and *ë_p_* → 0, from the error equation, we have *W^T^* (*q_m_*, *q̇_m_*)*θ̃* → 0.

**Theorem 2** (Persistent excitation condition)

With control laws (7), (11) and PAA (13), if the Gyroscope (5) is controlled to follow the reference Model (6) and *ω*_0_ ≠ *ω_f_*, the persistent of excitation condition is satisfied and all unknown gyroscope parameters are estimated correctly.

*Proof*:

By persistent excitation of *W*(*q_m_*, *q̇_m_*), we mean that there exist strictly positive constants *α*_1_, *T*_1_and *t*_0_ such that:

(15) $$\int_{t}^{t+T_{1}}W(q_{m},{\dot{q}}_{m})W^{T}(q_{m},{\dot{q}}_{m})d\tau \;\geq \;\alpha_{1}\;I\;\;\;\;\;\;\;\;\;\;\;\;\forall t\geq t_{0}$$

Since the Lyapunov derivative is negative semi definite, it can be argued that $\underset{t\to \infty }{\text{lim}}\tilde{\theta }={\tilde{\theta }}_{0}$, and if inequality (15) is satisfied, then:

$$\begin{array}{l} \alpha_{1}{\tilde{\theta }}_{0}^{T}{\tilde{\theta }}_{0}\;\leq \;{\tilde{\theta }}_{0}^{T}\underset{t\to \infty }{\text{lim}}{\int_{t}^{t+T_{1}}W(q_{m},{\dot{q}}_{m})W^{T}(q_{m},{\dot{q}}_{m})}^{}d\tau \;{\tilde{\theta }}_{0}\\ \;\;\;\;\;\;\;\;\;\;\;\;\;\;\;\;\;\;\;\;=\underset{t\to \infty }{\text{lim}}\int_{t}^{t+T_{1}}{\tilde{\theta }}_{0}^{T}W(q_{m},{\dot{q}}_{m})W^{T}(q_{m},{\dot{q}}_{m}){\tilde{\theta }}_{0}d\tau \\ \;\;\;\;\;\;\;\;\;\;\;\;\;\;\;\;\;\;=\int_{t}^{t+T_{1}}\underset{t\to \infty }{\text{lim}}\;{\tilde{\theta }}^{T}W(q_{m},{\dot{q}}_{m})W^{T}(q_{m},{\dot{q}}_{m}){\tilde{\theta }}^{}d\tau \end{array}$$

Since *W^T^* (*q_m_*, *q̇_m_*)*θ̃* →0, we get $\alpha_{1}{\tilde{\theta }}_{0}^{T}{\tilde{\theta }}_{0}\to 0$, and thus *θ̃* → 0 is achieved.

Because the trajectory *q_m_* contains two different frequencies as long as *ω*_0_ ≠ *ω_f_*, *α*_1_, *T*_1_ > 0 and *t*_0_ can always be found such that (15) is satisfied. According to the Theorems 1 and 2, trajectories *x*, *y* and their derivatives follow *x_m_*, *y_m_* and their derivatives respectively, and the estimates of gyroscope parameters converge to their true values. Consequently, a non-ideal Gyroscope (3) behaves like an ideal angle measuring gyroscope with the additional sinusoidal input *f_m_* as follows:

(16) $$\begin{array}{l} \ddot{x}+\omega_{0}^{2}x=2{\hat{\Omega }}_{z}\dot{y}+f_{xm}\\ \ddot{y}+\omega_{0}^{2}y=-2{\hat{\Omega }}_{z}\dot{x}+f_{ym} \end{array}$$

Note that control laws (7), (11) and PAA (13) are driven by reference model signals *q_m_*, *q̇_m_* and velocity measurement *q̇*. Since normally a velocity sensing circuitry produces a larger noise than position sensing [14], we introduce an adaptive observer to avoid measuring directly the velocity of the proof mass. In order to estimate velocity, we propose the following observer:

(17) $$\begin{array}{l} {\dot{\hat{q}}}_{p}={\hat{q}}_{v}+L(q-{\hat{q}}_{p})\\ {\dot{\hat{q}}}_{v}=-\omega_{0}^{2}{\hat{q}}_{p}-2\hat{\Omega }\;{\dot{\hat{q}}}_{p}+f_{m} \end{array}$$

where *q̂_p_* is the estimate of the position, ${\dot{\hat{q}}}_{p}$ is the estimate of the velocity, *q̂_v_* is an additional state of the velocity observer, and *L* is a observer gain matrix given by *L* = *diag*{*L*_1_, *L*_2_}. To complete the modification, the velocity term *q̇* in the adaptive control law and parameter adaptation laws given by Equation (11) is replaced by ${\dot{\hat{q}}}_{p}$, *i.e.*,:

(18) $$f_{0}=-\gamma_{1}({\dot{\hat{q}}}_{p}-{\dot{q}}_{m})$$

With the proposed observer, the velocity measurement based adaptive control structure is not modified, and the analytic results of stability and persistent excitation condition are preserved, which can be proved in a similar way as the one found in reference [11].

## 4. Angle Calculation

The response of Equation (16) contains both the auxiliary sinusoidal input *f_m_* and the angular rate signals, thus a demodulation process is required for extracting angle information from those signals.

If the line of oscillation of the proof mass with amplitude *M* is initially aligned with the *ê*_1_ axis of the inertial frame, then the steady-state response of the Equation (16) is given by:

(19) $$\begin{array}{l} x(t)=M\;\cos({\hat{\Omega }}_{z}t)\sin(\omega_{0}t)+B\;\sin(\omega_{f}t+\phi_{x})\\ y(t)=-M\;\sin({\hat{\Omega }}_{z}t)\sin(\omega_{0}t)+B\;\sin(\omega_{f}t+\phi_{y}) \end{array}$$

where:

$$\begin{array}{l} B=F_{0}\frac{{(\omega_{0}^{2}-\omega_{f}^{2})}^{2}+4\omega_{f}^{2}\Omega_{z}^{2}}{{(\omega_{0}^{2}-\omega_{f}^{2})}^{2}-4\omega_{f}^{2}\Omega_{z}^{2}}\\ \phi_{x}=\tan^{-1}\left(\frac{2\omega_{f}\Omega_{z}}{\omega_{0}^{2}-\omega_{f}^{2}}\right),\;\;\;\;\phi_{y}=\tan^{-1}\left(\frac{-2\omega_{f}\Omega_{z}}{\omega_{0}^{2}-\omega_{f}^{2}}\right) \end{array}$$

The rotation angle (*ψ* = Ω*_z_t*) can be calculated by demodulating process from the Equation (19) by multiplying these signals by sin(*ω*_0_*t*), and filtering the resulting signals with a low-pass filter. The demodulated rotation angle becomes:

(20) $$\psi =-{\text{tan}}^{-1}\left(\frac{\text{LPF}(y\;\text{sin}(\omega_{0}t)}{\text{LPF}(x\;\text{sin}(\omega_{0}t)}\right)-\psi_{0}$$

where *ψ*_0_ is initial procession angle and LPF denotes a low-pass filter.

**Figure 2.**
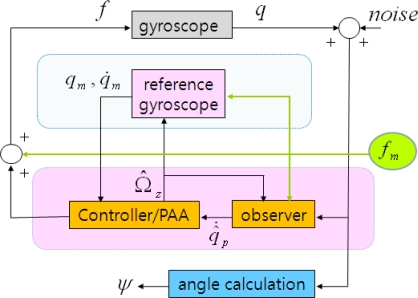
Proposed adaptive control scheme.

The bandwidth of the proposed controlled gyroscope is defined by the cutoff frequency of the low-pass filter used in the rotation angle calculation process. Considering typical bandwidth of angular rate is a few hundred Hz, the cutoff frequency of the low-pass filter can be chosen. The driving frequency *ω_f_* of the auxiliary input should be also carefully selected to be distant from the reference frequency *ω*_0_ of the ideal angle measuring gyroscope such that the difference of two frequencies is bigger than the cutoff frequency of the low-pass filter for successful separating rotation angle signal from the response to the auxiliary sinusoidal input. However, the driving frequency should not be too far apart from the reference frequency and also the magnitude of the auxiliary sinusoidal input *F*_0_ should not be chosen too small such that the magnitude *B* is large enough to make contribution to the persistent excitation condition. The overall block diagram of the proposed adaptive control scheme for an angle measuring gyroscope is shown in Figure 2.

## 5. Simulations

A simulation study is conducted to evaluate the proposed control scheme using the design data of the MEMS gyroscope model shown in [11]. The specified reference frequency is *ω*_0_ = 4.17*KHz*. For simulation purposes, we allowed that the natural frequencies of the x- and y-axis have ±15% deviation errors from the reference frequency, and the magnitude of coupled frequency and damping are ±1% variations in the magnitude of their nominal values. The position measurement noise is assumed to be zero-mean white with PSD of 1.49 × 10^−26^ m^2^. The auxiliary inputs are designed to be f_xm_ = f_vm_ = 343.24 sin(2π × 2.9 × 10^3^t)m/s^2^. The gyroscope parameters in the model and the numerical values for the controller in the simulations are summarized in Table 1. Note that these values are shown in non-dimensional units, which are non-dimensionalized based on length of one-microns and the reference natural frequency, 1/ω_0_ (s).

**Figure 3.**
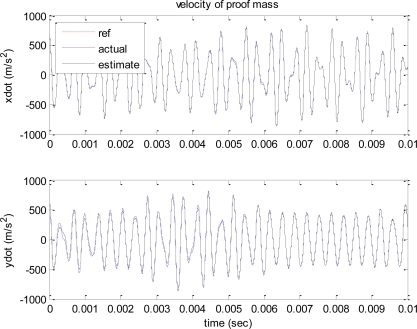
Velocity of proof mass.

**Figure 4.**
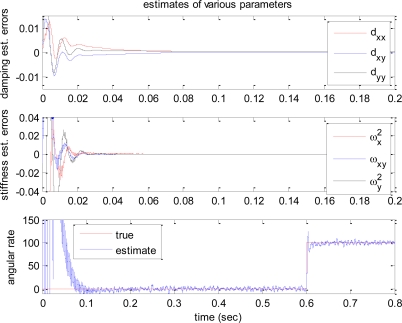
Time response of parameter estimation.

Figure 3 shows the reference, actual, and estimated velocity trajectories of the proof mass. All three values are almost identical, and the trajectory contains two different frequencies. Figure 4 shows the time responses of the estimation errors of the various gyroscope parameters.

**Figure 5.**
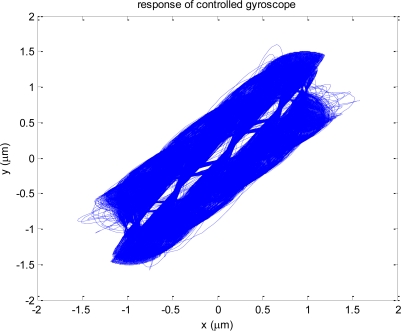
Trajectory of proof mass in x-y plane.

According to the plots, all estimation errors quickly converge to zero, and the estimate of angular rate also converges to its true value. Therefore dynamics of controlled gyroscope follows that of the ideal reference gyroscope. In these simulations, it is assumed that the gyroscope experiences step input angular rate of 100 deg/s at 0.6 s after the gyroscope is turned on. Figure 5 shows the trajectories of the proof mass in x-y plane. Compared to the response of the ideal angle measuring gyroscope shown in Figure 1, the precession of the proof mass is significantly disrupted due to the auxiliary control input *f_m_*. Thus a demodulation process is required for extracting angle information.

**Figure 6.**
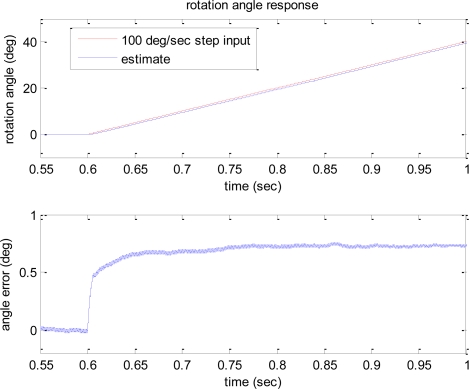
Time response of rotation angle estimate to the 100 deg/s step input.

**Figure 7.**
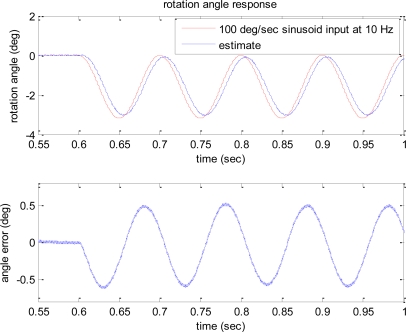
Time response of rotation angle estimate to the 100 deg/s sinusoidal input at 10 Hz.

The estimates of angle response to step and sinusoidal input angular rates of 100 deg/s are shown in Figures 6 and 7, respectively. The second plots in Figures 6 and 7 show the angle estimation errors.

According to the plots, the error bound of the angle estimation is 0.7 deg. Considering about 0.05 s delay introduced in the angle estimate due to the low pass filter in demodulation process, if the estimated angle is shifted 0.05 s and compared to the input angle, the angle estimation accuracy with the error bound of 0.2 deg can be achieved under the presence of noise. These simulation studies show the feasibility and effectiveness of the developed algorithm that is capable of directly measuring rotation angle without integration of angular rate.

## 6. Conclusions

This paper presents an adaptive control algorithm for realizing vibratory angle measuring gyroscope so that rotation angle can be directly measured without integration of angular rate, thus eliminating the accumulation of numerical integration errors. The proposed control algorithm uses a trajectory following approach. The reference trajectory that the gyroscope must follow is generated by an ideal angle measuring gyroscope driven by the estimate of angular rate and auxiliary sinusoidal input. The role of auxiliary sinusoidal input is to increase the complexity of the internal dynamics of the gyroscope so that its response is persistently exciting. In such a way, the developed control algorithm can identify and compensate for all fabrication imperfections and environmental variations in an on-line fashion. The stability of proposed adaptive controlled gyroscope is rigorously proved. An adaptive observer is also designed to avoid direct measurement of the velocity of the proof mass, since normally velocity sensing circuitry produces a larger noise than position sensing. The simulation studies show that the proposed control algorithm realizes angle measuring gyroscope operation successfully.

## Figures and Tables

**Table 1.** Non-dimensional values of the control parameters.

**Parameter**	Value
**gyroscope**	*ω*_0_ = 1, *ω_x_* = 1.05, *ω_y_* = 1.15, *ω_xy_* = 0,01, *d_xx_* = 1 × 10^−4^, *d_yy_* = 1.05 × 10^−4^, *d_xy_* = 0.01 × 10^−4^
**controller**	*γ*_1_ = 1, γ_R_ = 1/5, γ_D_ = 1/100, γ_Ω_ = 1/50
**observer / LPF**	*L* = *diag*{1, 1}, $\mathrm{LPF}={\left(\frac{0.05}{s+0.05}\right)}^{2}$

## References

[1] Yazdi N, Ayazi F, Najafi K (1998). Micromachined Inertial Sensors. Proc. IEEE.

[2] Painter C, Shkel A Detection of Orientation and Predicted Performance of a MEMS Absolute Angle Measuring Gyroscope.

[3] Park S, Horowitz R, Tan CW (2008). Dynamics and control of a mems angle measuring gyroscope. Sens. Actuat. A..

[4] Shkel A, Howe R, Horowitz R Modeling and Simulation of Micromachined Gyroscopes in the Presence of Imperfections.

[5] Friedland B, Hutton M (1978). Theory and error analysis of vibrating-member gyroscopes. IEEE Trans. Autom. Control..

[6] Shkel A, Howe R (2002). Micromachined Angle Measuring Gyroscope.

[7] Painter C, Shkel A (2005). Experimental Evaluation of a Control system for an absolute angle measuring micromachined gyroscope. IEEE Sensor. J..

[8] Piyabongkam D, Rajamani R, Greminger M (2005). Development of a MEMS gyroscope for absolute angle measurement. IEEE Trans. Control Syst. Technol..

[9] M’Closkey R, Vakakis A Analysis of a Microsensor Automatic Gain Control Loop.

[10] Leland R Lyapunov based Adaptive Control of a MEMS Gyroscope.

[11] Park S, Horowitz R (2004). New adaptive mode of operation for MEMS gyroscopes. ASME J. Dyn. Syst. Meas. Contr..

[12] Park S, Tan CW, Kim H, Hong SK (2009). Oscillation control algorithms for resonant sensors with applications to vibratory gyroscopes. Sensors.

[13] Slotine JJ, Li W (1991). Applied Nonlinear Control.

[14] Boser B Electronics for Micromachined Inertial Sensor.

